# A multimodal deep learning model to infer cell-type-specific functional gene networks

**DOI:** 10.1186/s12859-023-05146-x

**Published:** 2023-02-14

**Authors:** Shiva Afshar, Patricia R. Braun, Shizhong Han, Ying Lin

**Affiliations:** 1 Department of Industrial Engineering, University of Houston, Houston, TX 77204 USA; 2 Department of Psychiatry and Behavioral Sciences, Johns Hopkins School of Medicine, Baltimore, MD 21287 USA; 3 Lieber Institute for Brain Development, Baltimore, MD 21205 USA

**Keywords:** Cell-type-specific functional gene networks, Multimodal deep learning, Single-nuclei gene expression, Global protein interaction networks

## Abstract

**Background:**

Functional gene networks (FGNs) capture functional relationships among genes that vary across tissues and cell types. Construction of cell-type-specific FGNs enables the understanding of cell-type-specific functional gene relationships and insights into genetic mechanisms of human diseases in disease-relevant cell types. However, most existing FGNs were developed without consideration of specific cell types within tissues.

**Results:**

In this study, we created a multimodal deep learning model (MDLCN) to predict cell-type-specific FGNs in the human brain by integrating single-nuclei gene expression data with global protein interaction networks. We systematically evaluated the prediction performance of the MDLCN and showed its superior performance compared to two baseline models (boosting tree and convolutional neural network). Based on the predicted cell-type-specific FGNs, we observed that cell-type marker genes had a higher level of hubness than non-marker genes in their corresponding cell type. Furthermore, we showed that risk genes underlying autism and Alzheimer’s disease were more strongly connected in disease-relevant cell types, supporting the cellular context of predicted cell-type-specific FGNs.

**Conclusions:**

Our study proposes a powerful deep learning approach (MDLCN) to predict FGNs underlying a diverse set of cell types in human brain. The MDLCN model enhances prediction accuracy of cell-type-specific FGNs compared to single modality convolutional neural network (CNN) and boosting tree models, as shown by higher areas under both receiver operating characteristic (ROC) and precision-recall curves for different levels of independent test datasets. The predicted FGNs also show evidence for the cellular context and distinct topological features (i.e. higher hubness and topological score) of cell-type marker genes. Moreover, we observed stronger modularity among disease-associated risk genes in FGNs of disease-relevant cell types. For example, the strength of connectivity among autism risk genes was stronger in neurons, but risk genes underlying Alzheimer’s disease were more connected in microglia.

**Supplementary Information:**

The online version contains supplementary material available at 10.1186/s12859-023-05146-x.

## Background

Functional gene networks (FGNs) capture functional relationships among genes and provide a system-level understanding of gene function, that could further shed light on genetic mechanisms underlying human diseases. Each node in a gene network represents a gene and each edge represents a functional connection between a gene pair. Evidence of functional connections include gene co-expression, physical interaction of proteins, and text mining. Gene networks vary across tissues and cell types because some genes are functionally related only in certain tissues and cell types due to the specificity needed to give rise to an array of functions. However, most curated gene networks that have been developed, such as STRING [1] and HumanNet [2], incorporate evidence from many tissues, cell types, and organisms, and may not closely reflect the gene relationships within specific tissues or cell types. This may limit our understanding of gene functions and genetic mechanisms underlying human diseases that exist only in disease-relevant cell types.

On the other hand, computational approaches have been developed to predict gene interactions from gene co-expression patterns. The co-expression patterns of genes are usually quantified from gene expression signatures by using the statistical metrics such as Pearson correlation [3–6] and mutual information [7–9]. But predicting gene interactions from the single-cell gene expression data is challenging due to technical noise and the large amount of missing data [10]. The statistical metrics mentioned above, when applied to the single cell expression data, may only reflect chance or noise in the data. The recent advances in deep learning techniques have opened up new opportunities for in silico prediction of cell-type-specific FGNs. A convolutional neural network model (CNN) was recently developed to predict gene relationships in specific cell types through a novel encoding scheme that transforms pairwise gene expression data to an image-like co-expression matrix [11]. Although the CNN model provides a flexible computational framework and opportunity for further improvement by incorporating domain knowledge for a particular task, this approach still heavily relies on the quality of single cell gene expression data. In addition, it ignores the rich global functional information in the repositories of protein interaction networks, which limits their accuracy in gene interactions inference. Integrating the global functional information and single-cell gene expression data will lead to a comprehensive representation of gene interactions for more accurate inference of cell-type-specific gene networks. But only a few studies consider the integrative approach for predicting gene interactions at the cell type level [12–14].

In this study, we are interested in predicting cell type-specific gene functional relationships in the human brain. We reason that 1) the CNN framework is well suited to capture the 2D correlations in the image-like gene co-expression matrix; 2) integrating CNN framework with gene features derived from a global protein interaction network could learn a more comprehensive representation of genes and enable more accurate predictions of gene functional relationships. Accordingly, we developed a multimodal deep learning model (MDLCN) for predicting cell-type-specific FGNs by integrating single-nuclei expression data of the human brain with a global protein interaction network. We systematically evaluated the prediction performance of MDLCN and showed it outperformed the CNN and a conventional machine learning model (i.e. boosting tree model) that transforms the pairwise gene expression data to a correlation coefficient for predicting cell-type-specific FGNs. We further evaluated the cellular context of the predicted cell-type-specific FGNs through topological analyses of cell-type marker genes and genes underlying autism and Alzheimer’s disease.

## Methodology

**Fig. 1:**
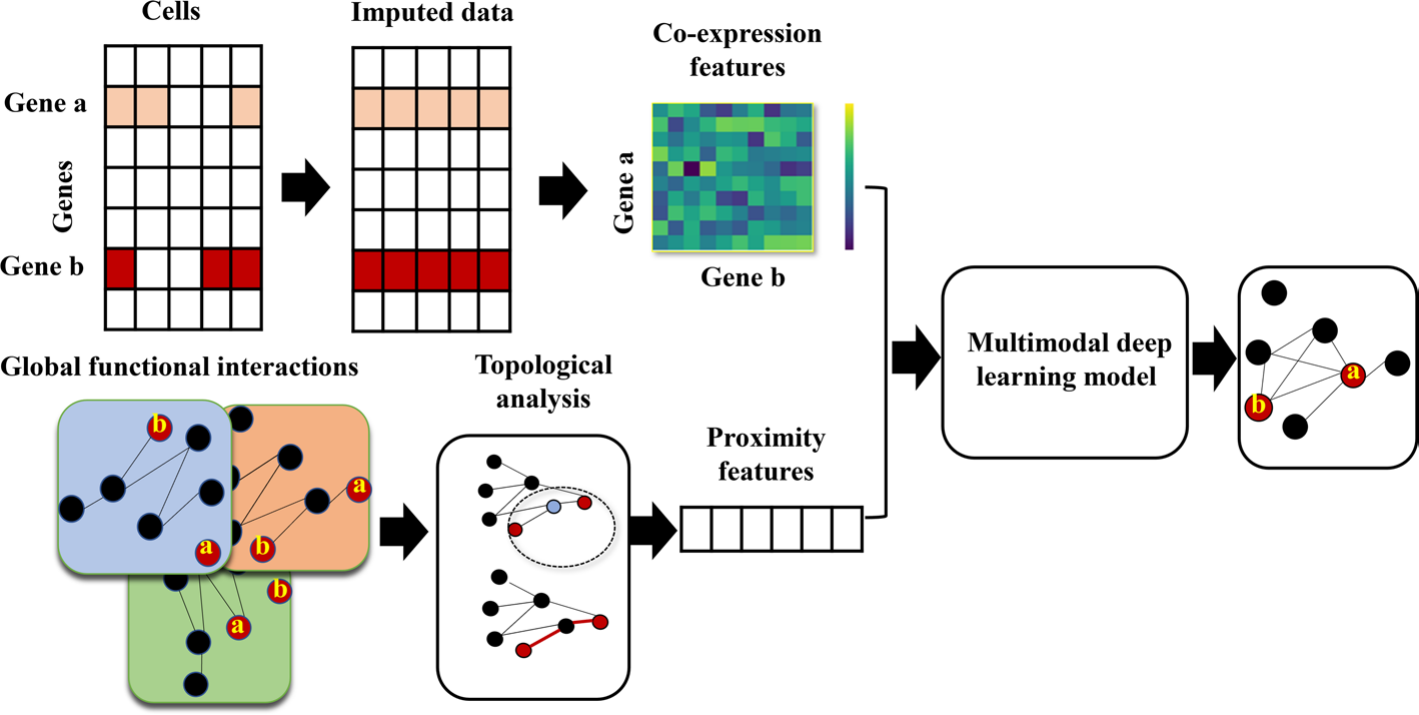
The framework of the proposed multimodal deep learning model for predicting cell-type-specific functional gene networks

We developed MDLCN, a multimodal deep learning model, for predicting cell-type-specific FGNs by leveraging single-cell gene expression data with a global protein interaction network (Fig. 1). Gene expression signatures of a gene pair were first transformed to a co-expression matrix that captures the joint density of co-expression patterns of the gene pair across the cells in a particular cell type. We computed a set of proximity features for each gene pair based on a global protein interaction network which we assembled from protein physical interaction evidence. The co-expression matrix and global proximity features of each gene pair were integrated to predict its functional relationship status through the MDLCN model. The MDLCN model was trained for each cell type using the cell-type-specific gold standards.

### Training dataset

We assembled a gold standard of gene pairs for building cell-type-specific FGNs following the approach for building tissue-specific FGNs in a previous study [14].We first constructed a cell-type-naive functional relationship gold standard from 564 expert-selected gene ontology (GO) terms and experimentally derived gene annotations. Gene pairs co-annotated to expert-selected terms were treated as positive examples (i.e. functionally related), and pairs not co-annotated to any of these terms were considered as negative examples. Next, we identified cell-type-specific genes that are defined as the top ranked genes by a specificity score that was computed by the average expression level of the gene in each cell type divided by the total expression values of the gene across all cell types [15]. The higher the specificity score of a gene in a cell type, the more specific the gene was to that cell type. Then, we combined the cell-type-naive gold standard with the cell-type-specific genes to construct the cell-type-specific gold standard.

Ultimately, our cell-type-naive gold standard included 3,619,063 positive gene pairs and 49,095,410 negative gene pairs. Table S1 presents the positive and negative class construction procedure (see Additional file 1). The positive gene pairs in our cell-type-specific gold standard were a subset of positive examples in the cell-type-naive gold standard, and the two genes of each pair were cell type-specific or one was cell type-specific and the other was a house-keeping gene as defined previously [16]. The negative gene pairs in our cell-type-specific gold standard were either: (1) positive examples in cell-type-naive gold standard, but one gene was specific to the corresponding cell type, and the other gene was specific to a different cell type; (2) positive examples in cell-type-naive gold standard, but one gene was specific to a different cell type, and the other gene was a house-keeping gene; (3) negative examples in cell-type-naive gold standard, but the two genes of each pair are cell type-specific or one was cell type-specific and the other was a house-keeping gene; (4) negative examples in cell-type-naive gold standard, but one gene was specific to the corresponding cell type, and the other gene was specific to a different cell type; (5) negative examples in cell-type-naive gold standard, but one gene was specific to a different cell type, and the other gene was a house-keeping gene.

The single-nuclei gene expression data used in this study includes 14,873 nuclei from the human brain that were clustered and annotated to major brain cell types in a previous study [17], including 3400 excitatory neurons, 1715 inhibitory neurons, 1897 astrocytes, 245 endothelial cells, 386 microglia, 2963 oligodendrocytes, and 682 oligodendrocyte precursor cells. We normalized the single nuclei count data by the "LogNormalize" function of Seurat that normalizes each feature count by the total counts in each cell, multiplied by a scale factor (10,000) and transformed to log scale [18]. Cell type-specific genes were the ones which ranked in the top 5% in the specificity scores for all cell types except for excitatory neurons for which the top 10% ranked genes were used so that a sufficient number of labeled gene pairs for model training could be collected. To have a balanced number of positive and negative gene pairs in each training set, the negative classes were randomly down sampled [19]. The number of positive and negative gene pairs in each cell type is presented in Table S2 (see Additional file 1).

### Transforming single-nuclei gene expression data to 2D co-expression matrices

We encoded single-nuclei gene expression data of the gene pairs to an image-like 2D co-expression matrix. Since the single-nuclei gene expression data suffers from technical noise and large number of missing values, we used a Markov affinity-based graph (MAGIC) method to impute the missing values and smooth the single nuclei expression data [10]. The MAGIC method shares information among similar cells using data diffusion to fill in missing gene expression values and restore the data structure accurately. Then, the range of the expression values of each gene was divided into $K$ equal bins. Then, for each pair of genes, 2D co-expression matrix was constructed by counting the number of cells that each gene expressed in the corresponding bins [11]. As the number of bins ($K$) plays an important role in the model performance in our experiments, it was tuned to 10, at which the model achieved the best prediction accuracy.

### Gene proximity features from global protein interaction network

Global protein interaction networks contain protein structural and functional information that are informative for cell-type-specific gene functional relationships. We assembled a global protein interaction network based on experimentally validated protein physical interaction evidence from multiple resources including Biogrid [20], IntAct [21, 22], APID [23] and Inweb [24].

After overlapping with genes in the single-nuclei gene expression dataset, the global protein interaction network contained 16,873 genes and 142,340,628 pairs of physical interactions. We used five metrics to measure the degree of similarity or proximity between a protein pair in the global protein interaction network, including Common Neighbors (CN), Jaccard’s Coefficient (JC), Preferential Attachment (PA), Adamic-Adar Coefficient (AA), and Path Distance (PD). These metrics measure different topological relationships between two proteins in the network. In particular, CN counts the number of common neighborhoods between the two proteins, JC quantifies the similarity between their neighborhoods, PA calculates the likelihood of link existence by measuring the strength of the hubness of the two proteins, AA computes the proportion of their shared links to the total number of their neighbors, and PD measures the length of the shortest path between the two proteins [25].

### Predicting cell-type-specific FGNs

**Fig. 2:**
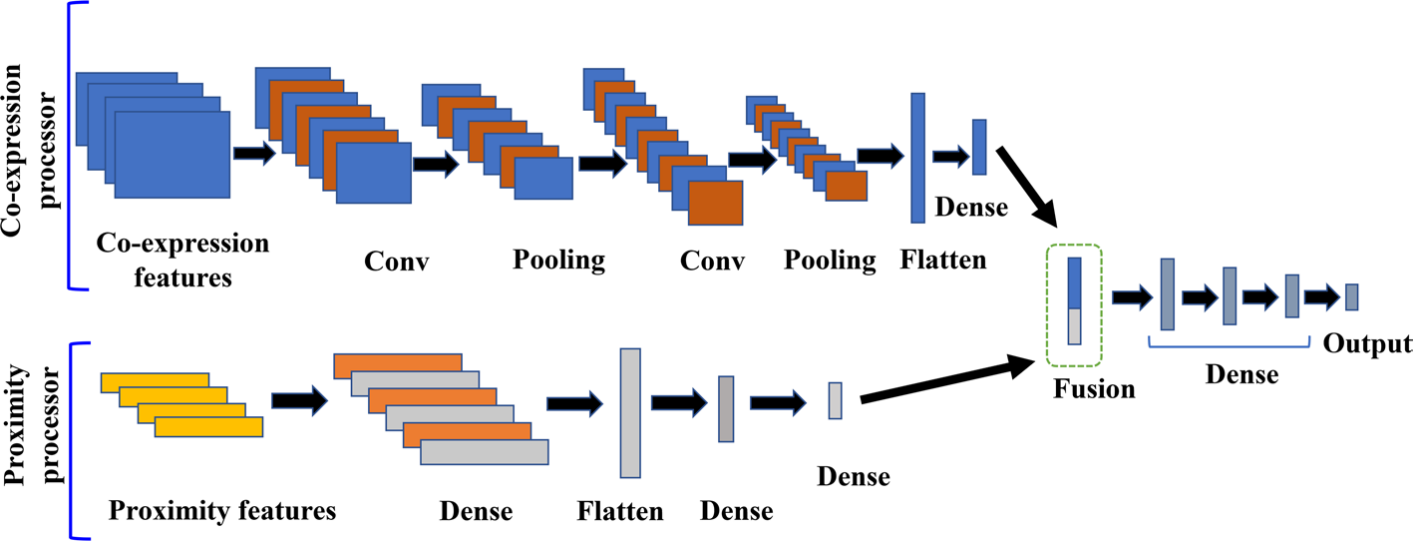
The architecture of multimodal deep learning model

We developed a multimodal deep learning model to predict cell-type-specific gene functional relationships from co-expression matrices and proximity features between two proteins in the global protein interaction network. For each pair of genes, the co-expression matrix and the vector of proximity features were exploited as two modalities in our model, including a co-expression-processor modality to extract representations from the co-expression matrix and a proximity-processor modality to extract representations from proximity features as shown in Fig. 2. In the co-expression-processor modality, the input layer is a co-expression matrix for each gene-pair. The modality consists of three convolutional layers which map the local conjunctions of features from previous layers to a feature map. Immediately after each convolutional layer, there is a max pooling layer which down samples the output of convolutional layers by taking the maximum value over an input window. At the end of this modality, a flattened layer is used to switch 2D features extracted from convolutional process to 1D features by retaining the weight orders and a densely connected layer is employed to compile the features extracted from previous layers to form the representations. The proximity-processor modality consists of an input layer for five proximity features between each gene pair, four densely connected layers and a flattened layer. The representations output from the two modalities are concatenated to a high-dimensional feature vector in a fusion layer and transformed through three densely connected layers. Finally, the feature vector is used in output layer to predict the probability of cell-type-specific functional connection between a gene pair. We used rectified liner activation function (ReLU) as the activation function across the whole network except the output layer where sigmoid function was used for binary classification. The dropout regularization is used in the multimodal deep learning model for preventing overfitting. The model was implemented using the Keras library in Python. We chose the Binary-Cross Entropy as the loss function and the Adam optimizer to update weights. The hyperparameters in the model, including the number of filters in the convolutional layer, the kernel size of the convolutional layer, the kernel size of the max pooling, the size of the dense layer in co-expression modality, the size of the dense layers in proximity modality, the position and rate of dropout, and the optimizer’s type, were tuned using the validation set and summarized in Table S3 in Additional file 1.

### Evaluation of model performance

We evaluated the performance of the MDLCN model in predicting functional relationships of gene pairs in three different test sets, including a 1) dependent test set that included gene pairs with both genes appearing in the training set; 2) partially dependent test set that included gene pairs with only one gene appearing in the training set; and 3) independent test set that included gene pairs with both genes not appearing in the training set. We used the area under ROC curve (AUC-ROC) and the area under Precision-Recall curve (AUC-PRC) as the evaluation metrics with five-fold cross validation. We compared the prediction performance of MDLCN with two baseline models. To evaluate the effects of the features from global protein interaction network, we compared the MDLCN with the single modality CNN model, which predicts the cell-type-specific FGNs from the 2D co-expression matrix only. The single modality CNN model consists of three convolutional layers which map the local conjunctions of features from previous layers, and after each convolutional layer there is a pooling layer that reduce the dimension of the output of convolutional layers to extract the essential information. At the end, there is a flattened layer which changes the 2D features extracted from convolutional process to 1D features by retaining the weight orders and a densely connected layer is used to compose the extracted features from previous layers to form the representations. To further demonstrate the advantages of using the 2D co-expression matrix compared to conventional statistical metrics, we also compared the MDLCN with a boosting tree model, which uses the Pearson correlation coefficients between gene expression signatures and the proximity features extracted from the global gene network as predictors. The boosting tree model is an ensemble of 150 decision trees that were built sequentially to improve the prediction accuracy of cell-type-specific FGNs.

### Topological analysis of cell-type marker genes

To evaluate the constructed cell-type-specific networks, we further examined whether cell-type marker genes have a distinctive topological structure across different cell-type-specific FGNs. Our rationales are (1) within each cell type, its marker genes have higher expression level and distinct functional role than the non-marker genes, leading to the higher hubness of marker genes in their associated cell-type-specific FGN; (2) the marker genes are cell-type specific which leads to higher topological score in their associated cell-type-specific FGN than in the other FGNs of not cell-type-specific. Cell-type marker genes were based on a previous study [26] and were defined as genes with at least one log-fold change in expression levels when cells of a given cell-type were compared against all other cells. The hubness of each marker gene was computed as the summation of edge weights that are directly connected to that gene. We also computed a topological specificity score for each marker gene to test whether cell-type marker genes have distinctive localization compared to random networks as done previously [12]. The topological specificity score represents the hubness of a gene in the predicted network normalized by its hubness distribution in random networks that were created by re-shuffling edge weights in the predicted network. The topological specificity score (topS) for each marker gene is calculated as topS = $\frac{{t}_{marker} -\mathrm{ m}({\mathrm{t}}_{marker}(\mathrm{g}))}{\mathrm{d}({\mathrm{t}}_{marker}(\mathrm{g}))}$, where ${t}_{marker}$ represents the summation of edge weights directly connected to marker gene, and ${t}_{marker}(g)$ represents the summation of edge weights directly connected to marker genes in random network $g$. The terms $\mathrm{m}({\mathrm{t}}_{marker}(\mathrm{g}))$, and $\mathrm{d}\left({\mathrm{t}}_{marker}(\mathrm{g})\right)$ indicate the average and standard deviation of ${\mathrm{t}}_{marker}(\mathrm{g})$ s for $g=1,\dots , 10$, respectively.

### Connectivity of disease genes in predicted cell-type-specific FGNs

To evaluate whether disease genes show cell-type-specific modularity in the constructed cell-type-specific networks, we assessed the connectivity strength between disease genes in each network. We considered two brain disorders: autism spectrum disorder (ASD) and Alzheimer’s disease (AD). We collected 408 high confidence ASD risk genes from the SFARI database [27] and 1,611 genes implicated in AD from the DisGeNET database [28]. We calculated the average connectivity over all pairs of disease genes for each network and compared the average connectivity with a background distribution from 1000 random gene sets matched by gene numbers and gene length with the disease genes. The Z-score was computed for each cell type with a large value of Z-score indicating that the disease genes show more significant cell-type-specific modularity in the corresponding cell-type-specific FGN. Specifically, Z-score was computed as Z-score = $\frac{{C}_{m} -\mathrm{ m}\left({C}_{m}\left(\pi \right)\right)}{\mathrm{d}\left({C}_{m}\left(\pi \right)\right)}$, where ${C}_{m}$ represents the average connectivity of disease genes, ${C}_{m}\left(\pi \right)$ represents the average of connectivity of random gene set with the same number of genes and same distribution of gene length as disease genes in random network $\pi$. The terms $m({C}_{m}(\pi ))$, and $d\left({C}_{m}\left(\pi \right)\right)$ represent the average and standard deviation of ${C}_{m}(\pi )$ s for $\pi =1,\dots , 1000$, respectively.

## Results

### MDLCN model performance

**Fig. 3:**
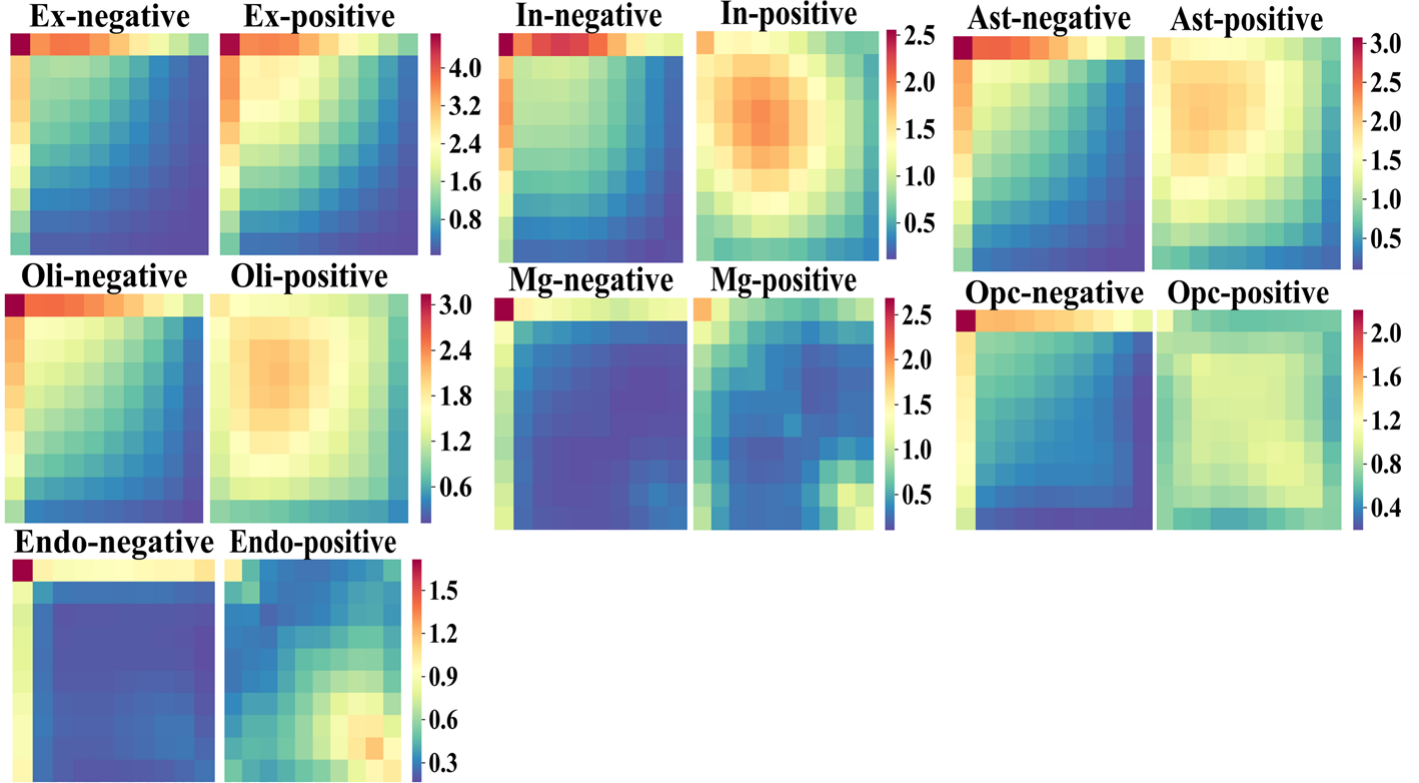
Co-expression heatmaps in positive and negative classes for excitatory neurons (Ex), inhibitory neurons (In), astrocytes (Ast), endothelial cells (Endo), microglia (Mg), oligodendrocytes (Oli), and oligodendrocyte precursor cells (Opc)

We first compared the 2D co-expression matrices between the positive and negative gene pairs for each cell type (Fig. 3). In all cell types, the positive gene-pairs show on average higher values in the bins corresponding to higher co-expression levels than the negative pairs, suggesting that the 2D co-expression matrices capture the complicated correlation among the genes to distinguish positive and negative classes. The proximity features extracted from global protein interaction network also show significant difference between the positive and negative gene pairs in all cell types, with the positive gene pairs having higher values for CN, JC, PA, and AA scores and having lower values in the PD score compared to negative class (see Fig. S. 1-Fig. S. 5, Additional file 1).

**Fig. 4:**
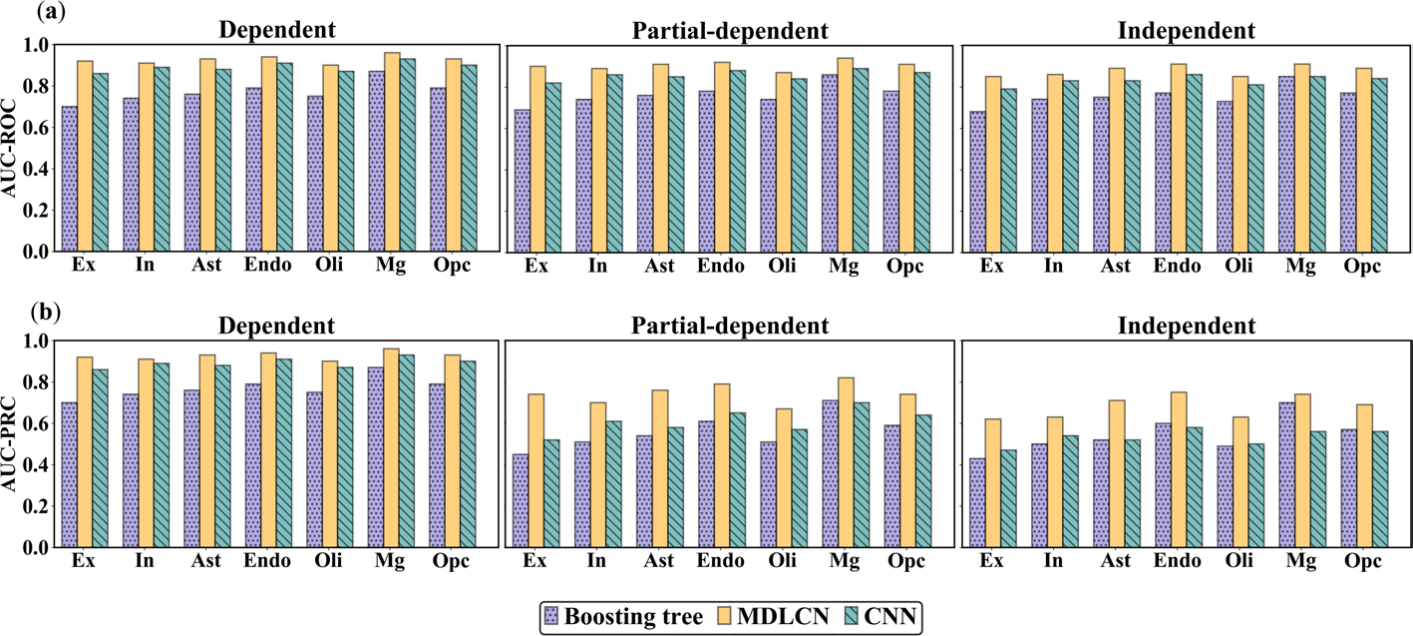
The (**a**) AUC-ROC and (**b**) AUC-PRC values obtained from Boosting-tree, CNN, and MDLCN models for three different test sets in excitatory neurons (Ex), inhibitory neurons (In), astrocytes (Ast), endothelial cells (Endo), microglia (Mg), oligodendrocytes (Oli), and oligodendrocyte precursor cells (Opc)

Prediction performance of the MDLCN model is illustrated in Fig. 4. The MDLCN model showed higher prediction accuracy in dependent, partially dependent, and independent test sets in all cell types compared to the CNN and boosting tree models. For example, the AUC-ROC achieved by the MDLCN model is around 15% higher than the boosting tree model and 4% higher than the CNN model in all three different testing scenarios. The improvements on AUC-PRC achieved by the MDLCN model are more substantial, suggesting the advantage of the model in identifying true cell-type-specific gene functional relationships.

For all the cell types, the prediction accuracy of the MDLCN model is satisfactory. The AUC-ROC (AUC-PRC) was higher than 0.90 (0.72) for the dependent test set, 0.87 (0.67) for the partially dependent test set, and 0.87 (0.63) for the independent test set (Fig. 4). The independent and partially dependent test sets consist of functional relationships among new genes, which were not seen in the training data set and more challenging to predict. Nonetheless, the MDLCN model still achieved good prediction performance in these test sets, which indicates that our model is promising in its ability to predict functional relationships between new genes.

### Downstream analysis

#### Topological analysis of cell-type maker genes

**Fig. 5:**
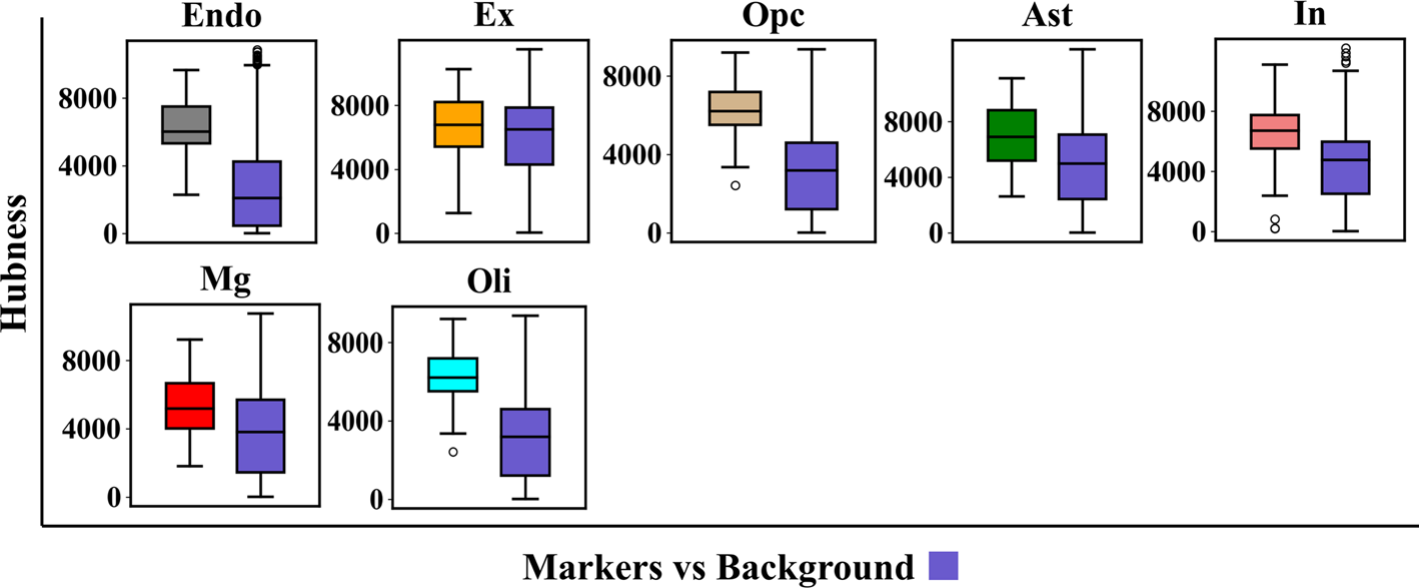
The hubness of marker genes versus non-marker genes in seven cell types: endothelial cells (Endo), excitatory neurons (Ex), oligodendrocyte precursor cells (Opc), astrocytes (Ast), inhibitory neurons (In), microglia (Mg), and oligodendrocytes (Oli). The P-values for even cell types are between ${10}^{-41}$ and ${10}^{-5}$

**Fig. 6:**
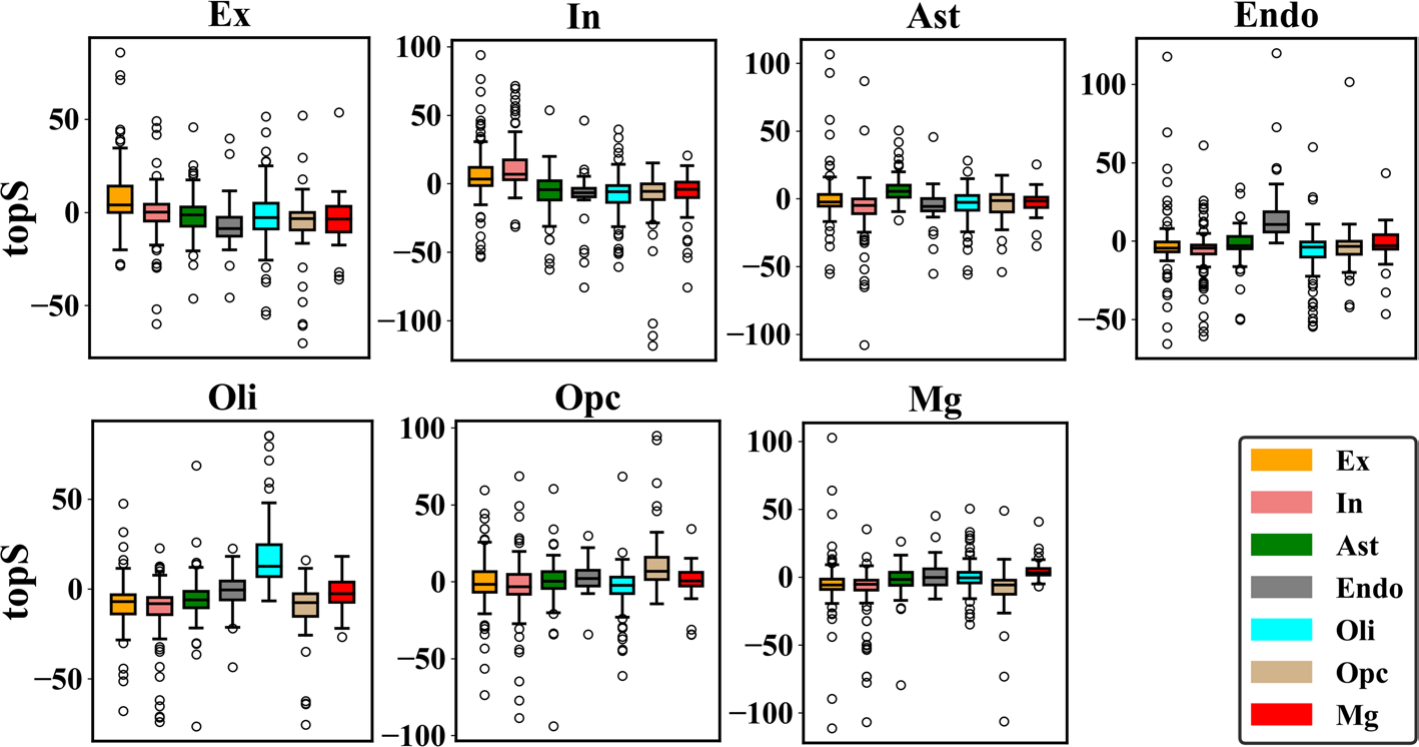
Topological specificity scores (topS) of marker genes in seven cell types: endothelial cells (Endo), excitatory neurons (Ex), oligodendrocyte precursor cells (Opc), astrocytes (Ast), inhibitory neurons (In), microglia (Mg), and oligodendrocytes (Oli). The P-values for seven cell types are between ${10}^{-10}$ and ${10}^{-4}$

We evaluated the topological features of cell-type makers genes in each constructed cell-type-specific FGN. We observed that marker genes had higher hubness than non-marker genes in the cell type that corresponds to the marker genes (Fig. 5). Furthermore, marker genes had higher topological specificity score in their corresponding cell type than the rest of the cell types (Fig. 6). These observations demonstrated that constructed cell-type-specific FGNs show distinct topological features for cell-type marker genes, reflecting the cellular context of predicted networks.

#### Connectivity of disease genes in predicted cell type-specific network

**Fig. 7:**
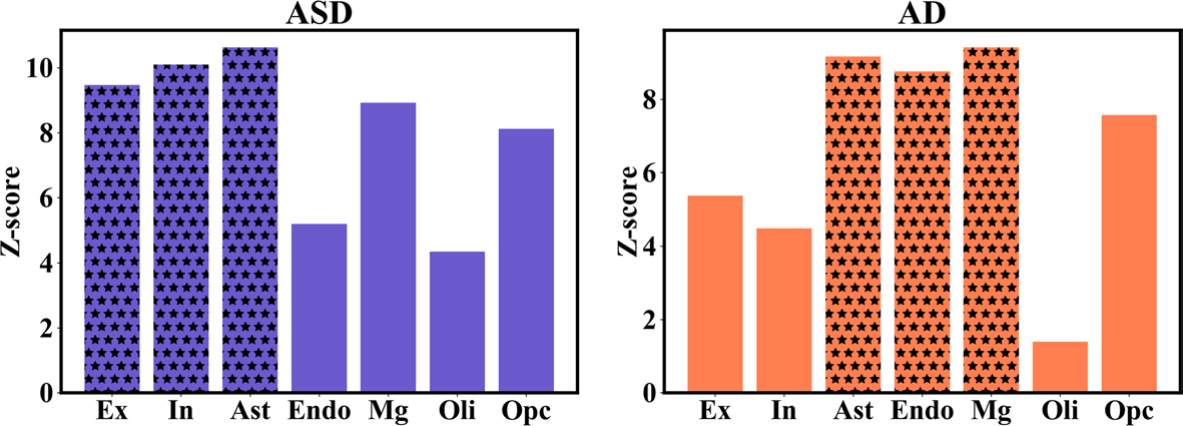
Z-scores for AD and ASD risk genes in endothelial cells (Endo), excitatory neurons (Ex), oligodendrocyte precursor cells (Opc), astrocytes (Ast), inhibitory neurons (In), microglia (Mg), and oligodendrocytes (Oli)

We compared the strength of connectivity among risk genes that underlie ASD and AD across different cell types (Fig. 7). We observed that disease genes tended to be functionally related in each cell-type-specific network, but the strength of connectivity among disease genes varied across different cell types. For example, the strength of connectivity among ASD risk genes is stronger in astrocytes, and neurons, and lower in microglia, endothelial cells, and oligodendrocytes. On the other hand, risk genes underlying AD were more connected in microglia, astrocytes, and endothelial cells, but less connected in neurons and oligodendrocytes. These results were consistent with the literature as ASD etiology is more related to the dysfunction of neurons and astrocytes [29], while microglia cells play a key role in AD pathogenesis [30], providing further evidence for the cellular context of predicted cell type-specific FGNs.

## Discussion and conclusions

We proposed a multimodal deep learning model MDLCN to predict cell type-specific FGNs by integrating single-cell gene expression data with global protein interaction networks. We showed the superior performance of the MDLCN model compared to both the CNN and boosting tree models. We further demonstrated evidence for the cellular context of predicted cell type-specific FGNs through the distinct topological features of cell-type marker genes and risk genes underlying two brain disorders: autism and Alzheimer’s disease.

While the MDLCN model holds the promise to predict functional gene relationships underlying a diverse set of cell types, the model should be viewed in light of two limitations. First, model performance may depend on the quality of the training dataset since the MDLCN employs a supervised approach. Second, the model can only predict the functional associations of two genes but not the direction of association. Further work includes extending the prediction to directional relationship of two genes by integrating more functional genomic datasets, such as those from ChIP-Seq or gene perturbation experiments.

## Supplementary Information

**Additional file 1:** Supplementary Tables and Figures.

## Data Availability

The protein physical interaction evidence was downloaded from multiple resources including Biogrid (https://wiki.thebiogrid.org/doku.php/biogrid_tab_version_2.0), IntAct (https://ftp.ebi.ac.uk/pub/databases/intact/current/psimitab), and APID (http://cicblade.dep.usal.es:8080/APID/init.action#tabr1). The single-nuclei gene expression data and the list of house-keeping genes are available at http://www.gtexportal.org/home/ datasets and https://m.tau.ac.il/~elieis/HKG/HK_genes.txt, respectively. The curated training data and cell-type-specific FGNs predicted from this study are available from the corresponding author on reasonable request.
