# Supplementary material for: A multimodal deep learning model to infer cell-type-specific functional gene networks
Source: BMC Bioinformatics. 2023 Feb 14;24:47. doi: 10.1186/s12859-023-05146-x (PMC9926713; doi:10.1186/s12859-023-05146-x)
Supplement: Supplementary file 1 — Additional file 1: Supplementary Tables and Figures. [file 12859_2023_5146_MOESM1_ESM.pdf]

Supplementary Tables and Figures

Table. S1. Training set construction process

<table>
<tr><td colspan="2" rowspan="2"></td><td colspan="2">Two genes of each pair were cell type-specific, or one was cell-type-specific and the other was a house-keeping</td></tr>
<tr><td>Yes</td><td>No</td></tr>
<tr><td rowspan="2">Positive examples in cell-type-naive gold standard</td><td>Yes</td><td>Positive pair</td><td>Negative pair</td></tr>
<tr><td>No</td><td>Negative pair</td><td>Negative pair</td></tr>
</table>

Table. S2. The number of gene-pairs in positive and negative classes in different cell types

Cell type	Number of gene pairs in positive class	Number of gene pairs in negative class
Excitatory neurons	54319	162957
Inhibitory neurons	32888	98664
Astrocytes	31772	95315
Endothelial cells	44611	133833
Microglia	43577	130731
Oligodendrocytes	26345	79035
Oligodendrocyte precursor cells	37493	112479

Table. S3. The hyperparameters of MDLCN

Number of filters in the convolutional layers	Kernel size of convolutional layers	Kernel size of max-pooling layers	Size of dense layer in co-expression modality	Size of dense layers in proximity modality	Size of dense layers	Drop-out rate
(32, 64, 128)	4×4	2×2	50	(5, 4, 3, 2)	(50, 20, 10)	0.1

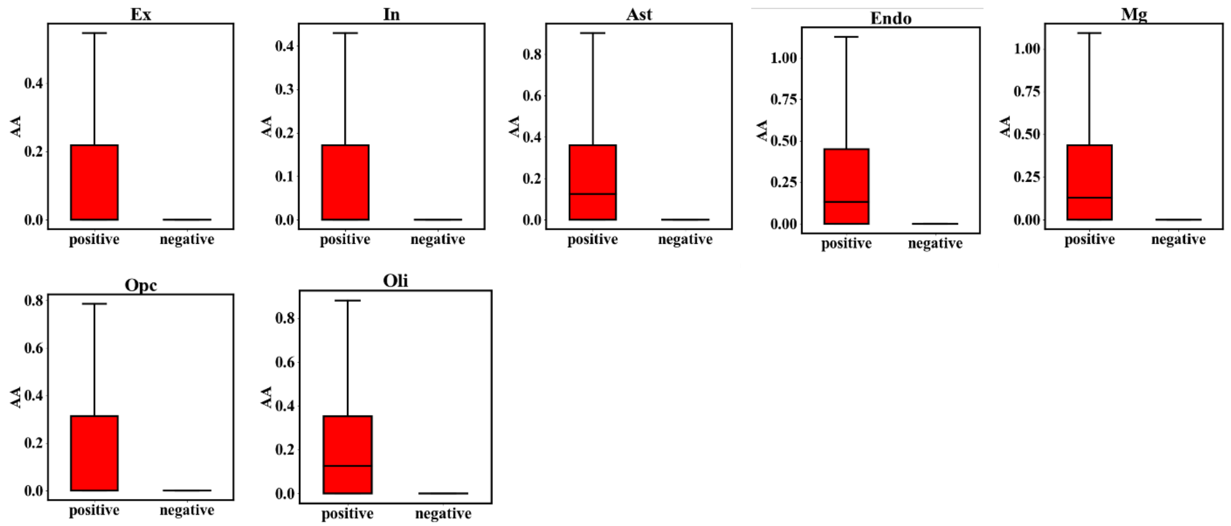

Fig. S. 1. Distribution of AA scores in positive and negative classes for excitatory neurons (Ex), inhibitory neurons (In), astrocytes (Ast), endothelial cells (Endo), microglia (Mg), oligodendrocytes (Oli), and oligodendrocyte precursor cells (Opc).

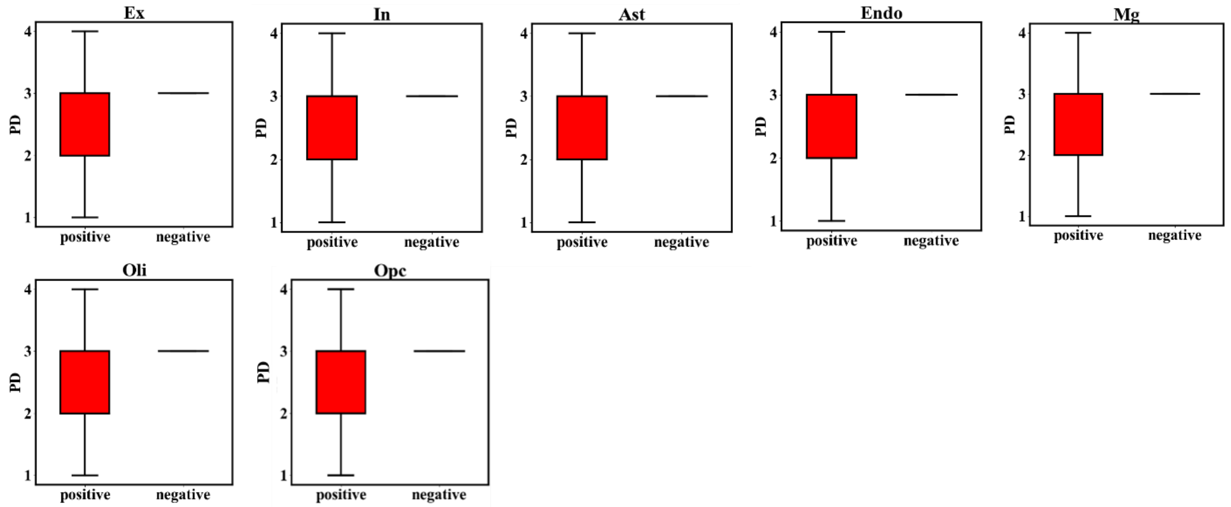

Fig. S. 2. Distribution of PD scores in positive and negative classes for excitatory neurons (Ex), inhibitory neurons (In), astrocytes (Ast), endothelial cells (Endo), microglia (Mg), oligodendrocytes (Oli), and oligodendrocyte precursor cells (Opc).

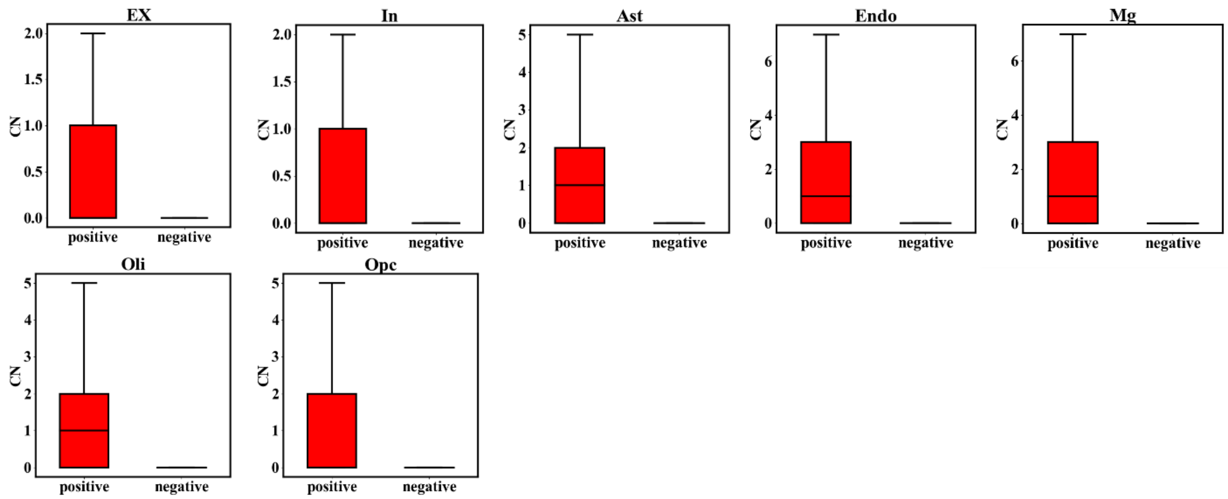

Fig. S. 3. Distribution of CN scores in positive and negative classes for excitatory neurons (Ex), inhibitory neurons (In), astrocytes (Ast), endothelial cells (Endo), microglia (Mg), oligodendrocytes (Oli), and oligodendrocyte precursor cells (Opc).

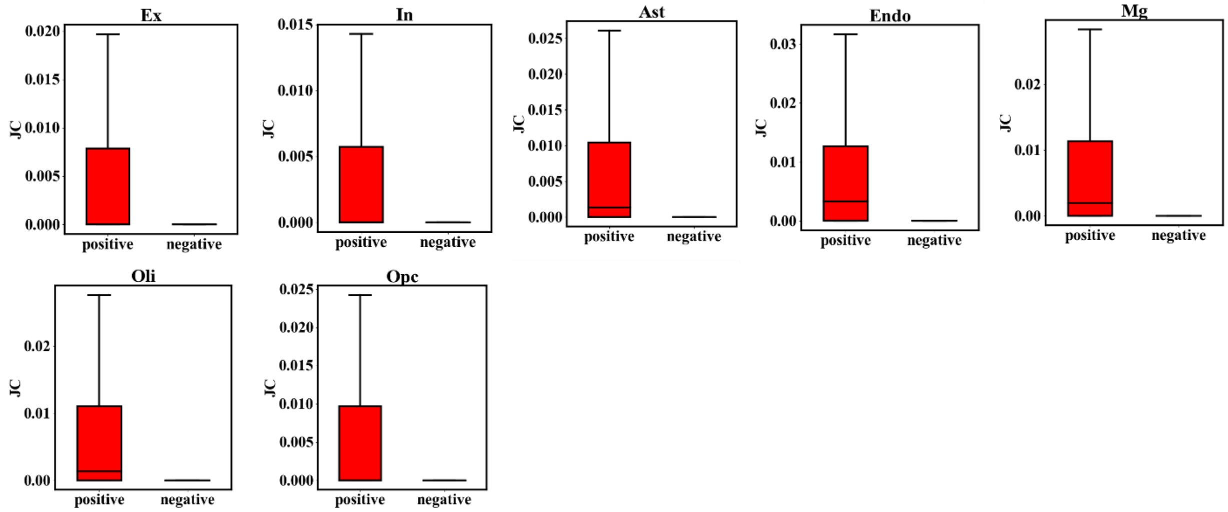

Fig. S. 4. Distribution of JC scores in positive and negative classes for excitatory neurons (Ex), inhibitory neurons (In), astrocytes (Ast), endothelial cells (Endo), microglia (Mg), oligodendrocytes (Oli), and oligodendrocyte precursor cells (Opc).

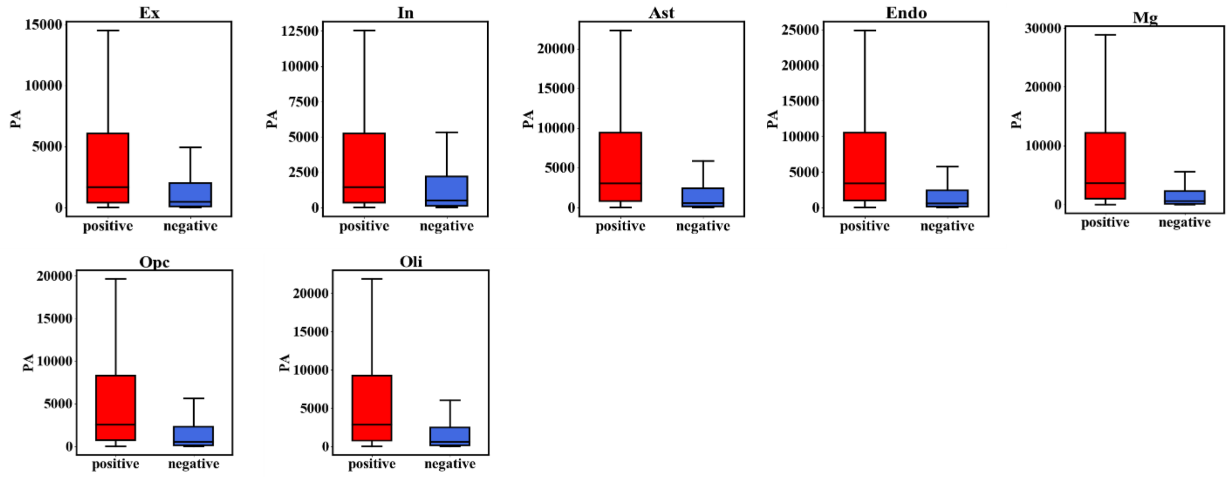

Fig. S. 5. Distribution of PA scores in positive and negative classes for excitatory neurons (Ex), inhibitory neurons (In), astrocytes (Ast), endothelial cells (Endo), microglia (Mg), oligodendrocytes (Oli), and oligodendrocyte precursor cells (Opc).
